# Does Exposure to Counterstereotypical Role Models Influence Girls’ and Women’s Gender Stereotypes and Career Choices? A Review of Social Psychological Research

**DOI:** 10.3389/fpsyg.2018.02264

**Published:** 2018-12-07

**Authors:** Maria Olsson, Sarah E. Martiny

**Affiliations:** Department of Psychology, UiT – The Arctic University of Norway, Tromsø, Norway

**Keywords:** role models, stereotypes, STEM, leadership, women, girls, counterstereotypical

## Abstract

Gender roles are formed in early childhood and continue to influence behavior through adolescence and adulthood, including the choice of academic majors and careers. In many countries, men are underrepresented in communal roles in health care, elementary education, and domestic functions (HEED fields, Croft et al., 2015), whereas women are underrepresented in the science, technology, engineering, and mathematical (STEM) fields (Beede et al., 2011) and top leadership positions (Leopold et al., 2016). Theories focusing on the development of gender roles suggest that across the lifespan people perceive certain roles to be more or less appropriate for their gender (e.g., Gender Schema Theory, Martin and Halverson, 1981; Social Role Theory, Eagly and Wood, 2011). Specifically, researchers have postulated that observing same-sex role models triggers learning processes whereby observers internalize gender-stereotypical knowledge of roles and act accordingly, which results in gender-congruent aspirations and behavior. It seems reasonable that if observing men and women in gender congruent roles fosters gender-congruent aspirations and behavior, then frequently observing gender-incongruent role models (e.g., male kindergarten teachers or female scientists and leaders) should reduce gender stereotyping and promote gender-counterstereotypical aspirations and behavior. In many countries, governments and societal decision-makers have formed initiatives based on the idea that exposure to gender-counterstereotypical role models influences aspirations and career choices among children, adolescents, and young adults. The present review gives an overview of research-based interventions involving observing or interacting with counterstereotypical role models, particularly focusing on outcomes for girls and women. Extending earlier reviews, we summarize laboratory-based and field-based studies and then critically discuss and integrate the findings in order to provide an overall picture of how counterstereotypical role models shape observers’ occupational aspirations and academic choices in childhood, adolescence, and young adulthood. We conclude by outlining suggestions for future research and briefly discussing implications for future interventions.

## Introduction

… relatable [female] role models will bring important future [female] scientists, mathematicians, technologists, engineers, innovators, and leaders into in the career pipeline.

1000 Girls, 1000 Futures

Gender roles concern the expectation of what conduct is appropriate for men and women based on the distribution of men and women in different roles (Eagly et al., 2000). Children from every walk of life are exposed to gender roles from an early age. First and foremost, children are exposed to gender roles in their immediate environment through their parents, siblings, relatives, neighbors, peers, and teachers, but also through educational resources, media, and popular culture. The social environment and media often depict traditional gender roles (Lauzen et al., 2008; Kahlenberg and Hein, 2010; Kan et al., 2011; Steyer, 2014; Koss, 2015; Murnen et al., 2016; Reich et al., 2018). For example, in many western countries, men spend more time in paid work whereas women spend more time in unpaid work (Kan et al., 2011). In addition, analyses of prime-time television programs show that men are typically represented in agentic (i.e., work-related) roles, whereas women are typically represented in communal (i.e., family related) roles (Lauzen et al., 2008). Given this widespread exposure to traditional gender roles, it does not seem surprising that children themselves report gender stereotypes, and gender-stereotypical ability beliefs, play preferences, peer preferences, and career aspirations from a very young age (Freedman-Doan et al., 2000; Levy et al., 2000; Serbin et al., 2002; Sebanc et al., 2003; Wilbourn and Kee, 2010; Baker et al., 2016; Bian et al., 2017; Golden and Jacoby, 2018). Specifically, research has shown that girls in 1st and 4th grade think the subjects they are worst at is computers and science, whereas boys think they are worst at reading (Freedman-Doan et al., 2000). Children’s gender-stereotypical beliefs of their current ability may shape their behavior later in life as they select activities they believe they are good at (Wigfield and Eccles, 2000).

One way that gender-stereotypical ability beliefs may become visible later on is in career choices. In many Western countries, men are underrepresented in communal roles in health care, elementary education, and domestic functions (HEED), whereas women are underrepresented in agentic and high-status roles such as leadership positions (Croft et al., 2015; Leopold et al., 2016), and in the science, technology, engineering, and mathematical (STEM) fields (Beede et al., 2011). There are several reasons why it is important to promote an equal representation of men and women in different occupational fields. First, gender equality provides benefits to both men’s and women’s welfare and health (Seedat et al., 2009; Read and Grundy, 2011; Holter, 2014). Second, increasing the number of women interested in STEM can meet the demands of an ever-expanding labor market and reduce the gender wage gap (Beede et al., 2011). Likewise, promoting men’s interest in HEED roles is important for overcoming labor shortages and promoting gender equality (Croft et al., 2015). Numerous initiatives and interventions have been implemented in several countries to encourage boys and girls to consider non-traditional occupational choices (e.g., Discover!; Little Miss Geek; 1000 girls, 1000 futures; Mind the Gap!; The Norwegian Government’s gender equality action plan; the WISE Campaign). These initiatives and interventions are often based on the rationale that observing or interacting with men and women in non-traditional domains, providing a so-called *gender-counterstereotypical role model*, will promote non-traditional behavior.

A gender-counterstereotypical role model is an individual who engages in a role that is antithetical to gender stereotypes (e.g., a female CEO, a female scientist, or a male preschool teacher). Role models have been defined in various ways in the literature (for an overview, see Morgenroth et al., 2015). We follow the lead of other researchers and consider role models as “individuals who influence [children’s, adolescents,’ and young adults’] achievements, motivation, and goals by acting as behavioral models, representations of the possible, and/or inspirations” (Morgenroth et al., 2015, p. 468). The present review focuses on interventions that utilize counterstereotypical role models to influence women’s aspirations to enter fields where they are underrepresented and negatively stereotyped. Role model interventions have been implemented with different goals in mind, such as promoting women’s interest and confidence in pursuing a career in STEM or other high-status roles such as top leadership and politics.

The underrepresentation of women in certain academic or high-status fields cannot be solely attributed to essential differences between men and women. First, mean gender differences in ability tend to be influenced by extreme cases at the end of the distribution (Hyde, 2005), and sometimes gender differences in aspirations and abilities only appear when gender stereotypes have been made salient (Spencer et al., 1999; Quinn and Spencer, 2001; Davies et al., 2005). Second, research suggests that at least part of the reason women do not enter certain academic or high-status fields originates in psychological barriers created by stereotypes. For example, a lack of females in STEM and top leadership positions may signal to women that members of their gender lack the skills necessary to be successful in these domains (Eagly et al., 2000). Thus, in order to encourage women to enter STEM and high-status positions where they are underrepresented and negatively stereotyped, it is important to expose women to female role models (Lockwood, 2006; Plant et al., 2009; Stout et al., 2011; but see Bagès and Martinot, 2011).

We will present literature on whether counterstereotypical role models have the potential to turn observers into role aspirants. Role aspirants are individuals who emulate and are inspired by role models (Morgenroth et al., 2015). Although the underrepresentation of men in certain educational and occupational domains certainly warrants empirical attention, we focus our review on girls and women because the vast majority of research has focused on women’s underrepresentation in male-dominated fields (for a discussion of the dearth of research on men in female-dominated HEED fields, see Croft et al., 2015). We will discuss wide-ranging studies exploring the effects of observing or interacting with gender-counterstereotypical role models from childhood to young adulthood including experimental research, correlational data, and evaluations of real-life interventions. Thus, extending earlier work, we will build a bridge between interventions conducted in the laboratory and interventions conducted in the field. We will also highlight factors that ought to be considered when developing future role model interventions. Role model interventions can encompass many different goals but are here defined as explicit attempts to change children’s, adolescents’, and young adults’ aspirations toward a gender-counterstereotypical occupational role by presenting them with a gender-counterstereotypical role model. In the following, we briefly summarize the main underlying theoretical assumptions about the effects of role models and then review the success of role model interventions in childhood, adolescence, and adulthood.

## Theoretical Underpinnings of Interventions

Although there is some disagreement amongst scholars regarding the underlying processes in the development of gender-congruent behavior, many theories have identified the observation of models–particularly same-sex models–as a major factor (e.g., Gender Schema Theory, Bem, 1981; Developmental Intergroup Theory, Bigler and Liben, 2006; Social Cognitive Theory, Bussey and Bandura, 1999; Social Role Theory, Eagly and Wood, 2011). It is not surprising then that many interventions that aim to target the underrepresentation of women in certain occupations and academic fields have involved exposure to stereotype-incongruent role models. It has been theorized that gender-stereotypical beliefs (which are widespread beliefs about the attributes of men and women, Heilman, 2001) are one of multiple factors that determine females’ achievement-related aspirations and choices (Wigfield and Eccles, 2000). While not all scholars agree that stereotypes play a major role in guiding gender-congruent behavior (e.g., Bussey and Bandura, 1999), some scholars argue that observational learning gives rise to stereotypical beliefs, which then foster stereotypical behavior through various mediating processes (Martin et al., 2002; Wood and Eagly, 2012).

Theories concerning the development of gender stereotypes and stereotype congruent behavior in childhood are very rarely applied to gender development in adulthood or vice versa (exceptions include Bigler and Liben, 2006; Wilbourn and Kee, 2010). Theories also differ in their terminology and emphasis on different cognitive processes. Nevertheless, some theories of gender development in childhood versus adulthood share the assumption that observational learning gives rise to stereotypical beliefs, which subsequently guide behavior (Gender Schema Theory, Bem, 1981; Social Role Theory, Eagly and Wood, 2011). For example, the assumption that children learn to associate men and women with certain attributes through observing their environment is a central tenet of Gender Schema Theory (Bem, 1981). This gender knowledge forms cognitive schemas, which give rise to stereotypical beliefs and influence behavior (Martin et al., 2002). According to Gender Schema Theory, a girl who chooses to play with a doll has engaged in the following thought process: dolls are “for girls” and “I am a girl” which means that “dolls are for me” (Martin and Halverson, 1981, p. 1120). If a gender-stereotypical environment fosters stereotypical knowledge, which in turn fosters stereotype congruent behavior, interventions involving exposure to gender-counterstereotypical role models should reduce gender stereotypes and enhance gender-counterstereotypical aspirations.

The assumption that adults’ stereotypes stem from observational learning is a key tenet of Social Role Theory (Eagly and Wood, 2011). According to Social Role Theory, people attribute the underlying cause of the unequal distribution of men and women in various roles to inherent gendered characteristics. Thus, because people mostly observe women in communal domains (where they are concerned with others, Abele and Wojciszke, 2007), people associate women with being socially skilled, nurturing, and caring. Likewise, because people mostly observe men in agentic domains (where they are concerned with pursuing their goals, Abele and Wojciszke, 2007), people associate men with being assertive and dominant. Men and women may subsequently internalize stereotypes about their gender, which guide their behavior (Hogg, 2000; Greenwald et al., 2002; Eagly and Wood, 2011). According to Social Role Theory, stereotypes are dynamic: when people perceive a non-traditional division of labor, they associate men and women with counterstereotypic characteristics (e.g., Diekman and Eagly, 2000; Wilde and Diekman, 2005). From this perspective, if the gender distribution of roles change, men’s and women’s gender stereotypes, self-concepts, and behavior should change accordingly. Thus, exposing men and women to counterstereotypical role models has the potential to change men’s and women’s aspirations and career choices.

Observational learning may operate differently at different stages of development. Notwithstanding this factor, it is possible to infer from theories applied in both childhood and adulthood that modeling is a precursor to the development of gender stereotypes (Gender Schema Theory, Bem, 1981; Social Role Theory, Eagly and Steffen, 1984). That being said, gender-developmental theorists and role-model theorists alike assert that role aspirants are far from passive learners (Martin et al., 2002; Bigler and Liben, 2006; Morgenroth et al., 2015). The effect of the role model on the role aspirant is instead moderated by the role aspirant’s previous experience, knowledge, and perceptions of the role model. The extent to which role models influence men’s and women’s aspirations and career choices may also interact with other factors such as direct instruction (Bussey and Bandura, 1999), parents’ differing perceptions of their sons and daughters (Furnham et al., 2002; Tenenbaum and Leaper, 2003), parents’ tendency to attribute their daughters’ success to hard work and their sons’ success to innate talent (Yee and Eccles, 1988; Räty et al., 2002), and biological sex differences (Eagly and Wood, 2013).

Because these theories propose that counterstereotypical role models influence child and adult role aspirants through the same processes, we review role model interventions that have been implemented from early childhood through early adulthood. Role model interventions have focused on a range of outcomes. Some interventions have targeted gender stereotypes, some have strived to promote self-efficacy and counterstereotypical behavior, and some have tried to enhance women’s aspirations toward fields where they are underrepresented. Role model research in childhood, adolescence, and adulthood has emphasized different outcomes, which means that we are not able to compare exactly the same variables at different developmental stages. For the childhood literature, we review studies that test the success of exposure to gender-counterstereotypical role models on girls’ gender stereotypes, aspirations, and behavior. For the adolescence and adulthood literature, we review studies that test the success of exposure to gender-counterstereotypical role models on girls’ and women’s gender stereotypes, self-concept, efficacy-beliefs (i.e., confidence in one’s abilities, Bandura, 1977), career aspirations, and academic choices.

## A Literature Overview of the Effects of Role Models in Early Childhood, Adolescence and Early Adulthood

In the following, we provide a comprehensive–but not exhaustive–overview of whether exposure to counterstereotypical role models influences children’s, adolescents’ and young adults’ gender stereotyping. In line with gender theories (Gender Schema Theory, Bem, 1981; Social Role Theory, Eagly and Wood, 2011), we argue that learning about gender is a process that takes place throughout a person’s lifespan. Exposure to or interaction with counterstereotypical role models may therefore influence role aspirants at every stage of development. Whereas research on exposure to counterstereotypical role models in adulthood has gained a lot of empirical attention over recent years, there has been a paucity of research on counterstereotypical role models in early childhood. In this review, we chose to include research spanning from early childhood into early adulthood, not because the literature easily lends itself to comparisons (in fact, it is quite the contrary!), but because we think that researchers and students interested in this topic would benefit from an overview. Previous research has tended to separate the study of gender in childhood from adulthood, which has resulted in different research foci in the two fields. Different research foci in childhood and adulthood literature can give the impression that learning about gender is vastly different across the lifespan. However, although adults and children may not be equally affected by observing or interacting with role models, the processes by which an adult learns is a continuation of processes by which a child learns. An overview can help to highlight both similarities and differences across the lifespan and potentially promote further research on role model processes in childhood.

An overview can also shed light on whether role model interventions are more effective in childhood or adulthood. Important and far-reaching decisions such as which classes to take in upper secondary school or at university are made during adolescence or early adulthood. Female participation in STEM subjects tends to diminish drastically at the secondary educational level and again at university (Cronin and Roger, 1999). This decrease suggests that the potential presence of psychological barriers at these educational stages demotivates adolescent girls and young women from pursuing careers in these fields. Role model interventions may thus be particularly critical during secondary and higher education. However, some scholars have argued that interventions aimed at changing stereotypes should take place in early childhood, preferably before children have developed a firm understanding of gender roles (e.g., Bigler and Liben, 2006). Early gender-stereotypical beliefs may shape children’s interests and have an accumulative effect on their skill acquisition and aspirations. Thus, interventions that occur later in development may be less effective or may have to be more comprehensive to counteract established interests and skills. Interventions may also be less successful once cognitive schemas are established, as schemas influence subsequent information processing (e.g., causing counterstereotypical information to be forgotten or distorted; Bigler and Liben, 1990; Frawley, 2008). However, interventions that take place too early may not be as effective as young children may not be able to generalize counterstereotypical information from one domain to another. This is because young children are more knowledgeable of stereotypical behavior among their own sex than they are of stereotypical behavior among the opposite sex. For example, although a young girl assumes that a child who plays with dolls also plays with a make-up kit, she may not assume that a child who plays with cars also plays with airplanes (Martin et al., 1990). Considering young children’s limited abilities in making logical inferences, interventions in early childhood may have to be more comprehensive than in adulthood as they have to model counterstereotypical behavior in many domains. These developmental factors support the need for an overview of how effective interventions have been at different stages in development.

## Effects of Exposure to Counterstereotypical Role Models in Childhood and Preadolescence

As children observe men and women in different roles, they learn what it means to be a man or a woman within their cultural context. Put differently, children form gender stereotypes based on their observation of role models. Role models that influence observers in one way or another have exerted a ‘role model effect.’ The majority of research-based interventions in childhood and preadolescence have focused quite broadly on promoting a broader repertoire of behaviors by exposing children and preadolescents to counterstereotypical role models. We will first review indirect evidence for the role model effect by summarizing studies that assess whether the stereotypicality of parents’ occupational roles correlate with the stereotypicality of their children’s occupational aspirations or behavior. We then turn toward direct evidence by summarizing experimental and non-experimental between-subjects design interventions.

### Correlational Evidence

Parents are the role models young children are exposed to most (Bandura and Bussey, 2004). In line with this, researchers have argued that parents’ occupations have a notable influence on offsprings’ gender stereotypes and career aspirations (e.g., Eagly et al., 2000). Numerous studies that have correlated mothers’ occupational roles with their daughters’ aspirations have found indirect evidence for the role model effect. For example, the stereotypicality of mothers’ work is associated with the stereotypicality of daughters’ occupational aspirations in both preschool and preadolescence (Marantz and Mansfield, 1977; Barak et al., 1991). In addition, daughters of mothers who work either full time or in counterstereotypical occupations also report more gender role flexibility in childhood, more counterstereotypical career plans in adolescence, more counterstereotypical behavior in adulthood, and less marriage-career-conflict concerns (Levy, 1989; Barnett et al., 2003; Fulcher and Coyle, 2011; Greene et al., 2013).

When interpreting these results, we have to keep several things in mind. First, all of the studies reported above have used a correlational design and therefore do not provide causal evidence for the role of observational learning in early childhood. Second, correlational relationships between parental occupational roles and children’s aspirations may, in some cases, be confounded with third variables such as instructional learning or how parents engage differently with their sons and daughters (Bussey and Bandura, 1999; Moon and Hoffman, 2008). Third, parental roles only account for small amount of variance in adults’ gender role attitudes (Barnett et al., 2003), and sometimes no significant relationship is found between mothers’ roles and daughters’ aspirations and behavior (Moen et al., 1997; Cunningham, 2001). Nevertheless, the findings reported above are important because they show that variations in gender roles within girls’ social reality can affect their aspirations and behavior. It is not surprising that the relationship between parents’ occupations and daughters’ gender-related aspirations and behavior is mixed, as many factors such as the mothers’ specific occupation and attitude toward work may influence daughters’ gender–related aspirations and behavior (Helms-Erikson et al., 2000). Taken together, the results of empirical studies investigating the relationship between parents’ occupational roles and daughters’ gender-related aspirations and behavior are mixed.

### Evidence From Interventions

In order to address the limitations of correlational designs and infer more conclusively the potential impact of role model interventions, it is important to review experimental research. Experimental interventions typically involve exposing children to counterstereotypical occupational role models for a relatively short period of time. Sometimes, interventions involve brief exposure that is repeated over several consecutive days. Occasionally, interventions involve exposure to counterstereotypical role models that span over several weeks or months. Studies that assess the effects of brief exposure to counterstereotypical role models are generally designed to assess the processes of observational learning, not the efficacy of role model interventions *per se*. Nevertheless, these studies provide useful information as many real-life interventions with counterstereotypical role models similarly involve only a brief exposure time. Following exposure to a counterstereotypical role model, children’s gender stereotypes and sometimes their aspirations or actual behavior are assessed. The majority of brief experimental interventions were conducted in or prior to the 1990s and not many recent studies in this area have been published. Much of the early research has already been summarized in several reviews (e.g., Katz, 1986; Liben and Bigler, 1987; Bigler, 1999). For this reason, we merely give a brief overview of this earlier work and integrate these findings with more recent findings in the subsequent section. We conclude by outlining the potential of role model interventions, and making suggestions for future interventions and research.

#### Do Children’s Gender Stereotypes Change Following Exposure to Counterstereotypical Role Models?

The methods used in role model interventions have typically consisted of exposing children to literature or commercials depicting men and women in counterstereotypical roles. In general, the literature shows that exposure to counterstereotypical role models influences girls’ gender-related beliefs. Among girls from preschool-age to 4th grade, exposure to counterstereotypical female exemplars reduced their occupational gender stereotypes and traditional attitudes toward women (Flerx et al., 1976; Ashby and Wittmaier, 1978; Pingree, 1978; Scott and Feldman-Summers, 1979; Trepanier-Street and Romatowski, 1999; but see Karniol and Gal-Disegni, 2009; Pike and Jennings, 2005). For example, Pingree (1978) presented 3rd graders with commercials that either depicted traditional women (e.g., a housewife) or non-traditional women (e.g., a female physician). Girls who had been exposed to non-traditional women reported less traditional attitudes toward women than girls who had been exposed to traditional women. Meeting counterstereotypical role models in real life also appear to reduce gender-stereotypical beliefs among children. Third graders reported less gender stereotypes after listening to men and women in counterstereotypical occupations talking about their careers (Tozzo and Golub, 1990). In addition, preadolescent girls were less likely to picture a scientist as male after interacting with female scientists during a 10-day long science camp (Leblebicioglu et al., 2011). Taken together, evidence shows that exposure to or interaction with counterstereotypical role models can reduce gender stereotyping.

#### Do Children Internalize Gender Stereotypes Following Exposure to Counterstereotypical Role Models?

Even though interventions involving exposure to counterstereotypical role models appear to change girls’ gender stereotypes, the overarching aim of role model interventions is not only to change specific stereotype beliefs but also to influence children’s subsequent behavior. It is therefore surprising that several of these studies have failed to include a measure of children’s aspirations or behavior (e.g., Tozzo and Golub, 1990; Trepanier-Street and Romatowski, 1999; Karniol and Gal-Disegni, 2009). The failure to include a measure of children’s aspirations or behavior may be due to a tendency among researchers to assume that boys and girls use gender stereotypes as a compass for behavior (Martin and Halverson, 1981). However, the assumption that stereotypes determine behavior is problematic. Research has repeatedly shown that changes in stereotypes do not reliably predict change in behavior (see Bigler, 1999). Specifically, studies have failed to find a significant change in girls’ aspirations for counterstereotypical occupations (Ashby and Wittmaier, 1978; Bailey and Nihlen, 1990; Bigler and Liben, 1990; Liben et al., 2001; Coyle and Liben, 2016) or preferences for counterstereotypical toys following a brief exposure to gender-counterstereotypical role models (Spinner et al., 2018, but see Ashton, 1983). Thus, the lack of correspondence between girls’ knowledge of what other women do and what *they* subsequently do suggests that stereotypes may not become internalized following short-term experimental interventions.

One factor that contributes to the lack of role model effects may be the extent to which the child perceives herself as similar to the role model. Anderson and Many (1992) analyzed 8- and 10-year-old children’s spontaneous thoughts on reading material that depicted children in non-traditional roles and found that the children sometimes struggled to relate to the counterstereotypical role models. Since role model effects are partly driven by role aspirants’ desire to become similar to the role model (Morgenroth et al., 2015), it seems crucial that the child identifies common ground with the counterstereotypical role model. Interventions that involve brief exposure to counterstereotypical exemplars may therefore benefit from explicitly highlighting similarities between the role model and the role aspirant to promote behavior change. Another factor that contributes to a lack of role model effects may be that children forget or distort counterstereotypical information, particularly if they are only briefly exposed to a counterstereotypical role model (Bigler and Liben, 1990; Frawley, 2008). Indeed, research has indicated that longitudinal interventions are more effective at eliciting changes. For example, Nhundu (2007) found that female primary school students who had been exposed to non-traditional educational material depicting females in non-traditional careers over a 3-year period expressed greater aspirations to pursue a non-traditional career than girls who had been exposed to traditional educational material. The education material explicitly encouraged young girls by including information such as: ‘Anybody can do any job they like as long as they get trained for it and become skillful.’ Thus, although this intervention was “successful,” it is not possible to establish whether the girls’ counterstereotypical aspirations were influenced by the repeated observation of counterstereotypical women, the direct encouragement, or a combination of these two factors.

#### Is the Role Model Effect Sustained and Does it Generalize to Other Domains?

Although children sometimes appear to internalize counterstereotypical information following exposure to counterstereotypical role models (e.g., Ashton, 1983), one must not assume that role model effects observed immediately after a brief exposure will be sustained. First, observations of behavior at one time point are not reliable indicators of permanent behavioral change in young children (Green et al., 2004). Second, stereotype change recorded immediately after an intervention is not always observed at a 1-week follow-up (Flerx et al., 1976; Savenye, 1990). This might be the case because children are exposed to traditional gender role information in their everyday life, which might overwhelm the effect of the intervention. The majority of studies, however, have failed to assess whether stereotype change following brief exposure to counterstereotypical role models is sustained. Thus, in order to draw firm conclusions regarding the longevity of role model effects following brief exposure to counterstereotypical exemplars, more research that assesses children’s gender stereotyping, aspirations, and behavior at several time points following the intervention is needed.

Moreover, it is questionable whether brief exposure to counterstereotypical role models in one domain will influence what is considered gender-appropriate in another domain. Research suggests that if change in stereotyping is observed at all, it is limited to the specific domains modeled in the intervention. For example, 3rd and 4th grade students read eight stories over a 4-week period either depicting a majority of males or a majority of females engaging in traditionally masculine roles. Children who had read about counterstereotypical women reported less stereotypical beliefs about women, but only for the roles that were portrayed by the characters in the stories (Scott and Feldman-Summers, 1979). The limited potential for counterstereotypical role models to eradicate traditional gender role beliefs may be determined by cognitive abilities, which preclude young children from making generalizations to other domains (Bigler and Liben, 1992). However, Trepanier-Street and Romatowski (1999) found stereotype change for occupations that were not included in the intervention. Children from three different preschools read six books over the course of 2 months that depicted both children and adults in counterstereotypical occupational roles. After listening to the stories, children engaged in several activities (e.g., children participated in a group discussion or listened to an adult talking about their career). It is thus possible that children reported less gender stereotypes for domains that were not included in the reading because they had also engaged in discussions about other occupational gender roles. Liben and Bigler (1987) also point out that although the abovementioned intervention was successful, the activities varied for each preschool and it therefore remains difficult to evaluate exactly which factor caused the effects and how to replicate them.

Evaluations of studies involving longitudinal exposure to counterstereotypical exemplars suggest that interventions focusing solely on targeting gender roles in one domain may not cause children to alter their gendered behavior in other domains. For example, Nhundu (2007) found that although girls’ stereotypes about occupations and their occupational aspirations appeared less gender-traditional following exposure to counterstereotypical occupations, girls still embraced gender roles relating to domestic work and emphasized the importance of women prioritizing family over career. Thus, despite a positive effect on girls’ career aspirations, girls’ sense of the priority of domestic work for women may counteract these effects. Interventions must therefore be comprehensive and must target gender stereotyping more broadly than the occupational domain. Moreover, it may also be important for interventions to influence not only the role aspirant, but also her family and peers (Adler et al., 1992). Research on an affirmative action program promoting females into leadership positions in local communities showed that counterstereotypical role models who are observable by the entire community influence not only the behavior of the role aspirant but also those of the wider community (Beaman et al., 2012). Specifically, in communities where there had been more than one period with a female leader, girls reported more educational aspirations, better educational outcomes, and less responsibility for domestic tasks, and parents reported higher career expectations for their daughters. Thus, when the entire community is exposed to female role models, it may make it easier for girls to choose non-traditional paths.

To summarize, brief exposure to counterstereotypical role models appear to change children’s gender stereotypes on a short-term basis. However, the changes in stereotypes are not always sustained and do not necessarily affect children’s aspirations and behavior. These modest role model effects are not surprising given that the exposures to counterstereotypical exemplars in experimental interventions are brief and might stand in sharp contrast to what the children experience and observe in their everyday life when observing their parents or consuming media. Having said that, we conclude that based on the current literature it would be premature to dismiss the potential of brief exposure to counterstereotypical role models on children’s aspirations and behavior. More research is needed to assess not only if, when, and why changes in stereotyping are sustained and internalized, but also whether changes in stereotyping have ‘spill over effects’ to other domains not present in the interventions. To our knowledge, no research to date has assessed how early exposure to counterstereotypical role models influences girls’ later career choices. However, women sometimes attribute their motivation to pursue academic studies to a female role model they were exposed to early in life (Lockwood, 2006). It thus seems reasonable that small changes in interests in early childhood can set the child on a different trajectory that may accumulate into counterstereotypical behavior later on. While it appears that longitudinal exposure to counterstereotypical role models may change children’s aspirations, the extent to which changes in aspirations in childhood are realized later on in adulthood is not clear. This is because there is a tendency for role model interventions to focus on gender stereotypes in one domain (e.g., the occupational domain) and not address gender expectations in other domains (e.g., the domestic domain). This may be problematic as some girls may see the home domain and the work domain as mutually exclusive. Due to greater exposure to female role models in the domestic domain than in the occupational domain, expectations to engage in the domestic role (e.g., to look after children at home) may be greater than expectations to engage in the agentic role (e.g., to pursue a high-status career). This means that even though girls may express counterstereotypical occupational aspirations following exposure to counterstereotypical exemplars, these aspirations may clash with gender expectations in the domestic domain later in life, which may preclude girls from pursuing high-status careers. In order for role model interventions to have the predicted effect in adulthood, interventions ought to confront the expectation that women will serve as the primary caregiver by also exposing girls to males engaging in the domestic domain.

### Future Research on Interventions in Childhood

The aim of reviewing interventions in early childhood was not only to evaluate these interventions, but also to identify potential for new research. One implication of this review is that it is not clear whether role model effects are driven by children’s propensity to emulate same-sex role models (Bussey and Bandura, 1999), or because counterstereotypical role models lead children to change the way they see themselves (Martin et al., 2002). Thus, future research on interventions should assess gender stereotypes, self-stereotyping, and subsequent behavior to determine whether a change in stereotypes is internalized and acted upon. This could potentially be assessed by observing children’s behavior over a long period of time and using child-friendly implicit measures to assess stereotypes (e.g., Green et al., 2004; Most et al., 2007; Banse et al., 2010). Implicit measures may sometimes be preferred over explicit measures as implicit measures are less dependent on young children’s ability to report their inner beliefs accurately and less susceptible to social desirability bias. A second future direction derives from the finding that children as young as 3 years old hold stereotypes about communal behavior (Baker et al., 2016). Thus, future research should assess whether children are able to infer communal and agentic traits from counterstereotypical role models, if they internalize them, and whether this influence a range of behaviors and preferences that were not necessarily targeted in the intervention. In addition, although it has been found that self-efficacy beliefs predict preadolescents’ career choices (Bandura et al., 2001), there is to our knowledge no research on whether exposure to counterstereotypical role models influences young children’s self-efficacy beliefs. Finally, more research should evaluate existing field-based interventions.

Based on theoretical reasoning, we proposed that observing or interacting with counterstereotypical role models would change children’s gender stereotypes and their sense of self. The research reviewed above only partially supports this claim. More research is needed to draw firm conclusions about the impact of counterstereotypical role models on role aspirants, and to integrate other processes that shape girls’ aspirations and behavior.

## Effects of Exposure to Role Models in Adolescence and Early Adulthood

We now move our focus from childhood and preadolescence to adolescence and early adulthood. Many role model interventions in adolescence and early adulthood are based on the same underlying principle as in early childhood and preadolescence. Namely that observers internalize gender-stereotypical knowledge of roles and act accordingly, which results in gender-congruent aspirations and behavior. Interventions in adolescence and young adulthood are typically more focused on a specific domain than in childhood and preadolescence. The ultimate goals of interventions in this age-group are to influence girls’ and women’s academic aspirations and career-related choices, especially focusing on domains where women are underrepresented and negatively stereotyped. To provide a justification for role model interventions, we first review correlations between the number of female role models in non-traditional fields and non-traditional role aspirants. We then turn to direct evidence by summarizing interventions that involve brief exposure to a counterstereotypical role model in the laboratory, and brief or prolonged interactions with a counterstereotypical role model in real life. We finish by outlining recommendations for future research.

### Correlational Evidence

If the proportion of female role models corresponds to the proportion of female role aspirants in non-traditional fields, then it provides prima facie evidence that the role models have influenced observers’ achievements, motivation, or goals. There is correlational evidence for the role model effect in several domains where women are underrepresented, including politics, science, and engineering (Sonnert et al., 2007; Wolbrecht and Campbell, 2007). For example, adolescent girls talk more about politics and report more future intentions to engage politically in countries where there is a greater number of female politicians (Wolbrecht and Campbell, 2007). Moreover, research that has looked at the relationship between the number of counterstereotypical role models and the number of counterstereotypical role aspirants at United States universities over time has found that if the percentage of female faculty members in a science and engineering department increases by 10%, the percentage of female majors in biological sciences, physical sciences, and engineering can be expected to increase by 1.2% (Sonnert et al., 2007). The small effect sizes reported may seem to suggest that having more same-sex role models has little relevance to achieving overall gender equality. However, considering the cumulative impact small effects can have in real life over the course of time, these results should not be overlooked (Eagly, 1996). In addition, although the role model effect appears to be small, the effect is more pronounced in the presence of more than one gender-incongruent role model (Nixon and Robinson, 1999; Campbell and Wolbrecht, 2006; Sonnert et al., 2007; but see Canes and Rosen, 1995).

However, it is not possible to infer causal relationships from cross-sectional findings. It could be that a stronger presence of female role models encourages the participation of female role aspirants due to a role model effect or it could be that the corresponding increase in both female role aspirants and female role models is caused by a third unknown variable. Thus, despite promising evidence from correlational studies, experimental or between-subjects design studies are needed to make causal inferences about the impact of gender-incongruent role models on role aspirants.

### Evidence From Interventions

The role model literature in adolescence and adulthood has gained attention in recent years. Experimental laboratory studies have typically involved providing female university students with information about women who are successful in fields where women are underrepresented and negatively stereotyped. Field-based between-subjects design studies have typically assessed the effect of interacting with female counterstereotypical role models. Following exposure to counterstereotypical role models, the extent to which girls or women have internalized the characteristics, behavior, or goals of the role model is assessed. In the following, we review interventions that involve exposure to or interaction with counterstereotypical role models from a broad range of academic or career-related settings. We focus exclusively on interventions in domains where women are underrepresented and negatively stereotyped. We propose that counterstereotypical female role models modify existing knowledge about women, which becomes internalized by the role aspirant, and this internalized knowledge then enhance self-efficacy beliefs, aspirations, and performance.

#### Do Adolescents’ and Adults’ Gender Stereotypes Change Following Exposure to Counterstereotypical Role Models?

One aim of role model interventions using counterstereotypical role models is to change girls’ and women’s perceptions of what they themselves can or should do by changing perceptions of what women in general can do. Studies have shown that students presented with descriptions or portrayals of non-traditional women changed their stereotypes about women, at least temporarily (Savenye, 1990; Dasgupta and Asgari, 2004; Rosenberg-Kima et al., 2008). For example, Dasgupta and Asgari (2004) presented female students with pictures and descriptions of several famous women in leadership positions in counterstereotypic fields such as science, business, law, and politics. Female students subsequently took part in an Implicit Association Test (Greenwald et al., 1998), which assessed the strength with which they associated women and men with being leaders and supporters. The results showed that female students were quicker to associate women with leadership following exposure to counterstereotypical women. This effect was replicated in a longitudinal design that took advantage of the pre-existing differences in the proportion of female faculty at two universities. These findings suggest that exposure to counterstereotypical exemplars can reduce gender stereotypes.

#### Do Adolescents and Adults Internalize Gender Stereotypes Following Exposure to Counterstereotypical Role Models?

Brief exposure to just one counterstereotypical female role model in STEM can also enhance, at least temporarily, female role-aspirants’ self-efficacy beliefs, determination to succeed, and performance in domains where women are underrepresented and negatively stereotyped (Marx and Roman, 2002; McIntyre et al., 2003; Rosenberg-Kima et al., 2008; Plant et al., 2009; Stout et al., 2011; Shin et al., 2016). The theoretical reasoning that underlie many role model interventions is that women see themselves in line with prevailing stereotypes (Guimond et al., 2006). From this follows that if a woman starts to perceive women in general as more agentic, she should also view herself as more agentic. In other words, following exposure to gender-counterstereotypical information, role aspirants should see themselves in less stereotypical ways. However, only a handful of studies have assessed the extent to which brief exposure to counterstereotypical role models causes women to internalize counterstereotypical information (also known as self-stereotyping, Guimond et al., 2006).

Several studies show that the way adult women see themselves change following brief and long-term exposure to counterstereotypical female role models (e.g., Lockwood, 2006; Asgari et al., 2010; Stout et al., 2011; Shin et al., 2016). However, not all role model interventions include a measure of gender stereotypes (e.g., Marx and Roman, 2002), and those that do sometimes fail to find a role model effect on gender stereotypes (Plant et al., 2009; Stout et al., 2011; Shin et al., 2016). For example, Plant et al. (2009) found that although middle-school girls reported greater self-efficacy and greater interest in engineering-related careers after being exposed to female engineers, they still endorsed traditional gender stereotypes related to engineering-related fields. Thus, the evidence as to whether the role model effects reported above were facilitated through a change in gender stereotypes and corresponding self-stereotyping remains inconclusive.

#### Is the Role Model Effect Sustained and Does it Generalize to Other Domains?

Adolescents and adults appear to internalize counterstereotypical information immediately following brief exposure to counterstereotypical exemplars. However, since the majority of laboratory-based studies have failed to use a follow-up design, it is not possible to affirm whether brief exposure to counterstereotypical role models has an enduring effect on role aspirants’ academic performance and career-choices (but see Herrmann et al., 2016). It seems likely that interactions over a long period of time with a counterstereotypical role model have more substantial role model effects than a brief exposure. To address the decreasing proportion of women in advanced STEM courses, several field-based interventions have been implemented during foundational STEM courses. They have found that female students exposed to female role models are more likely to set high-achieving goals and take intermediate courses in their respective fields than those exposed to only male role models (Asgari et al., 2010; Carrell et al., 2010; Porter and Serra, 2017). This role model effect is only observed in subjects where females are underrepresented, which indicates that female professors, rather than being better teachers than male professors, help to break down some of the psychological barriers preventing women from pursuing certain fields (see also Carrell et al., 2010). Thus, it seems that longitudinal exposure to counterstereotypical role models has the potential to enhance the effects reported by studies on short-term exposure. However, we cannot conclude from these studies that female professors affected role aspirants by challenging gender stereotypes. For example, it could be that the female professors facilitated a climate in which female students felt more comfortable actively participating, which had an effect on their performance, and ultimately their aspirations.

For role models to change how role aspirants see themselves, it may not be enough for female role aspirants to become aware that other women have achieved success in a given domain. It may also be critical that the role aspirant see themselves as similar to the role model (e.g., Rosenberg-Kima et al., 2008; Cheryan et al., 2011; Stout et al., 2011; Asgari et al., 2012; Hoyt et al., 2012). For example, Rosenberg-Kima et al. (2008) exposed undergraduate students to either a relevant role model (young and cool) or an irrelevant role model (old and uncool). Female students reported more self-efficacy if they had been exposed to a relevant role model than if they had been exposed to an irrelevant role model. Feelings of similarity are important because they convey the “if she can, so can I” idea to the role aspirant, which facilitates gender-counterstereotypical self-stereotyping. Interventions that fail to facilitate identification with the role model may not result in a role model effect. Studies that have assessed interventions in which adolescent girls engaged in science tasks and interacted with female scientists revealed that girls did not immediately and spontaneously view the female scientists as potential role models (Buck et al., 2008; O’Brien et al., 2017). Specifically, girls only began to view the female scientists as role models after establishing personal connections with them (Buck et al., 2008). Thus, it may be necessary for interventions to allow girls to establish personal bonds with the role model to facilitate aspirations toward a domain, particularly among younger girls who are not already invested in STEM. To highlight similarities between role aspirants and role models, some initiatives have tried to make female counterstereotypical role models more relevant by feminizing them. One example of this is the Science Cheerleaders initiative. In this initiative, girls who pursue science also do cheerleading at public events. The goal of this initiative is to reduce negative stereotyping about female scientists. To our knowledge, there has been no scientific evaluation of the Science Cheerleaders initiative. However, research suggests that employing *highly* feminine role models may be unsuccessful and even backfire. For example, Betz and Sekaquaptewa (2012) found that 6th and 7th grade girls who did not strongly identify with STEM reported less self-efficacy, less current interest in math, and less aspirations to pursue math after being exposed to a highly feminine role model in STEM. The feminine role model failed to produce a role model effect because the observers viewed the combination of femininity and success in STEM to be unachievable.

Taken together, brief exposure may inadvertently deter role aspirants from fields where they are underrepresented and negatively stereotyped because of two reasons. First, role aspirants see very successful women as exceptions to the rule and therefore not representative of their group (Kunda and Oleson, 1995). Second, role aspirants fail to see themselves in the role model (Rudman and Phelan, 2010; Hoyt and Simon, 2011). For example, Hoyt and Simon (2011) found that after reading about successful female leaders, female undergraduate students not only gave themselves worse evaluations on a leadership task but they also perceived the task as more difficult. This is because observing a counterstereotypical role model may result in a contrast-effect whereby the role aspirants think they cannot achieve the same level of success as the role model (also known as upward comparison threat, Rudman and Phelan, 2010). This is contrary to an assimilation-effect where observers’ performance improves following exposure to a successful gender-incongruent role model (Latu et al., 2013). Firm conclusions on why brief exposure to counterstereotypical role models appear to sometimes cause contrast-effects and sometimes cause assimilation-effects cannot be drawn by comparing the design of existing studies. However, it seems that a role model effect is less likely to occur when the role aspirants perceive themselves as unable to achieve what the role model has achieved (Lockwood and Kunda, 1997). For example, when undergraduate women had made an incremental attribution, i.e., when they believed that successful women had achieved success through hard work, discipline, and persistence, they were more likely to associate themselves with leadership traits than when they had made an entity attribution, i.e., when they believed successful women had achieved success because of their talent (Hoyt et al., 2012). This suggests that in order for female counterstereotypical role models to be effective role models and reduce stereotypical beliefs about women’s capabilities, it is important that female counterstereotypical role models are seen as representative of women in general.

The research reviewed above suggests that brief and longitudinal exposure to counterstereotypical role models can change women’s gender stereotypes and self-stereotyping. Moreover, exposure to or interaction with counterstereotypical role models can enhance role aspirants’ immediate self-efficacy beliefs and performance, and even influence role aspirants on a long-term basis by affecting their academic choices. While exposure to counterstereotypical role models appears to break down some of the psychological barriers to women’s participation in, or aspirations toward, fields where they are underrepresented, it is not always possible to determine whether changes in self-stereotyping are responsible for these role model effects. Thus, more research is needed to identify when and to what extent changes in self-stereotyping underlie role model effects. The cause of role model effects is interesting from both a theoretical and practical point of view. If the presence of female role models facilitates active participation in class, for example, then active participation may be important for enhancing feelings of self-efficacy and spurring interest toward domains where women are underrepresented and negatively stereotyped (but see Weisgram and Bigler, 2007). If stereotypes drive role model effects, then interventions should focus more actively on challenging stereotypical beliefs about women. Such interventions may benefit from carefully selected role models as similarity between role aspirants and role models seems crucial to facilitate self-stereotyping (McCrea et al., 2012).

### Future Research on Interventions in Adolescence and Adulthood

One of the goals of this review was to identify challenges and limitations in the role model literature for future research to address. Although numerous studies involving counterstereotypical role models have been conducted, they have been conducted with different goals in mind, with samples that are either partly invested or not invested in the role models’ field of expertise, and within different academic fields (for an exception, see Shin et al., 2016). This provides a number of questions for future research. First, research should address whether exposure to counterstereotypical role models promotes the same degree of counterstereotypical aspirations in all fields where women are underrepresented and negatively stereotyped. Second, research is needed to explore in greater detail what psychological processes drive these effects. Third, research must systematically assess how interventions are affected by role aspirants’ current interest or investment in the field. Fourth, future research must take a more holistic view to incorporate the role of the wider community (e.g., family, peers, or romantic partners) in depressing role model effects. Lastly, empirical research is needed to assess the efficacy of addressing gender roles in domains that seem incompatible with pursuing a career in a high-status field (e.g., marriage-career conflicts, childrearing) for longitudinal success.

Based on theoretical reasoning, we examined empirical support for the notion that observing or interacting with counterstereotypical role models would change adolescent’s and adult’s self-stereotyping. The research reviewed above only partially supports this claim. More research is required to establish the role of self-stereotyping in role model effects.

## Discussion

The current unequal distribution of women in various occupational roles acts as a psychological barrier to women’s entry into certain academic and high-status professional fields. In other words, occupational gender roles are both an antecedent to, and a consequence of, gender congruent behavior. Many initiatives that aim to promote women’s entry into fields where they are underrepresented and negatively stereotyped are based on the notion that this can be achieved through exposure to counterstereotypical female role models. The main aim of this review was to infer from correlational, laboratory-based, and field-based studies the potential of counterstereotypical role models to promote girls’ and women’s aspiration toward counterstereotypical occupational roles by counteracting the endless stream of gender-stereotypical information children, adolescents, and young adults are faced with on a daily basis.

First, we established that long-term exposure to counterstereotypical role models (e.g., mothers in non-traditional work, female politicians, and female faculty) in role aspirants’ natural environment positively correlated with their aspiration toward, and engagement with, counterstereotypical roles. Second, we assessed whether these role model effects could be simulated by time-limited role model interventions and, if so, what processes drive these role model effects. Our review of the role model literature showed that brief exposure to counterstereotypical role models in both childhood and adulthood is sometimes able to change stereotypical beliefs about women, at least temporarily. Despite this, we found that role aspirants-particularly young children did not always internalize characteristics of the role models. On the one hand, it is possible that brief exposure to counterstereotypical role models in early childhood is not sufficient to shift the way young girls perceive themselves. On the other hand, is possible that the lack of reported role model effects in early childhood are attributed to the limited number of times internalization has been assessed. We initially set out to provide an overview of interventions in childhood, adolescence, and adulthood in order to draw conclusions about what kinds of role model interventions are more effective in early childhood or later in development. However, the limited number of studies on how role models’ influence children’s aspirations and behavior means it would be premature to draw firm conclusions at this point. Third, we assessed whether long-term exposure to counterstereotypical role models generated more pronounced role model effects. We identified that longitudinal interventions, particularly those that involved the community, follow-up activities, or explicit encouragement, appeared to have an effect on children’s and preadolescents’ aspirations and behavior. Similarly, longitudinal exposure that facilitated active engagement appeared to enhance role model effects among young adults, particularly among highly motivated students. In comparison to role model research in adolescence and adulthood, role model research in early childhood and preadolescence has not assessed whether factors such as perceived dissimilarity suppresses role model effects. In adolescence and adulthood, it is clear that gender-counterstereotypical role models must challenge existing gender stereotypes, but at the same time not be seen as *too* atypical. Taken together, the reviewed literature suggests that interventions that aim to promote counterstereotypical behavior can be effective at any point in a person’s lifespan but should be designed with the role aspirants in mind, considering their current interests and motivations to engage in that behavior.

## Potential for Future Role Model Interventions

The underlying reason for why some role model interventions are “successful” is not always clear. Most field-based studies in childhood, adolescence, and adulthood have involved observational learning, active engagement, and sometimes instructional learning (e.g., Jayaratne et al., 2003). The question as to whether role model effects are reliant on both exposure to and interactions with counterstereotypical role models, or whether role model effects can be facilitated by observational learning alone warrants attention. This is important to assess since interventions that utilize mere observations of role models are potentially more cost-effective than interventions that require interactions with counterstereotypical role models over a long period of time (Herrmann et al., 2016). Moreover, there is no evidence to support the hypothesis that children’s self-stereotypes change following exposure to counterstereotypical role models. As such, the role model effect observed in childhood may be driven by imitation processes (Social Cognitive Theory, Bussey and Bandura, 1999) rather than by self-stereotyping processes (Gender Schema Theory, Martin et al., 2002). Future research should thus address through what pathway role model effects in childhood occur so this can be directly addressed in interventions.

Although research has not established that mere exposure to counterstereotypical role models promotes counterstereotypical behavior and aspirations in early childhood, several large-scale initiatives have been developed based on this idea. For example, Norway is seeking to recruit more male preschool teachers under the assumption that exposure to men in communal roles will reduce gender stereotyping and promote non-traditional occupational choices among children (see Norwegian Government’s Gender Equality Action Plan, 2014). While this initiative has not yet been empirically evaluated, qualitative analyses of children’s perceptions of male preschool teachers have found no evidence that daily exposure to counterstereotypical role models (i.e., male preschool teachers) challenges or changes children’s stereotypes. First, gender does not appear to be a notable factor in preschool children’s descriptions of their male teacher (Sumsion, 2005), meaning that children may not learn to associate men with communal behavior. Second, analyses have suggested that children observe their male preschool teacher as someone who typically engages in stereotypical behavior (e.g., Sumsion, 2005; Harris and Barnes, 2009). For example, Sumsion (2005) found that children never depicted their male preschool teacher engaging in traditional ‘female’ play but frequently depicted him as heroic and resourceful, as someone engaging in traditional ‘male’ play. Thus, based on the findings from these qualitative studies, one might conclude that exposure to counterstereotypical role models (although intended to reduce stereotyping) may sometimes inadvertently reinforce traditional gender roles. However, in our opinion, these conclusions should be treated with caution. It might be the case that specific conditions need to be met in order to ensure that male preschool teachers are perceived as role models. For example, preschoolers might need to be exposed to more than one counterstereotypical role model in order to generalize the communal behavior they observe in their male teachers to men in general.

More assessments of real world interventions are needed. One factor that should be considered is how the change in stereotypes is measured. Interventions are sometimes deemed successful based on a change in explicit stereotypes (e.g., Leblebicioglu et al., 2011). This could be problematic as research has shown that exposure to counterstereotypical role models enhance women’s self-concept and performance through implicit rather than explicit stereotypes (Dasgupta and Asgari, 2004). Second, it is important to consider changes in a range of domains, even those that were not directly targeted in the intervention. Interventions that focus primarily on stereotypes in the occupational domain may not be comprehensive enough to facilitate real change in girls’ future career choices because they do not also target gender roles in the domestic domain. Domestic expectations are present early on and may conflict with counterstereotypical aspirations. Thus, in order to demonstrate to girls that pursuing a career and raising children are not mutually exclusive, future interventions may benefit from portraying a female role model who has both a successful career and children. The risk of this approach is that female role models who manage to excel in both occupational and domestic roles may be seen as achieving unattainable success. Future interventions thus need to take care to present relatable role models whose success appears attainable. In order to reduce expectations that women will take the bulk share of domestic work, it may also be important to conduct interventions with boys. Without a corresponding shift in boys’ attitudes toward communal roles (Sinno and Killen, 2009), girls may be unlikely to pursue high-status or demanding careers due to difficulties with pursuing a career while simultaneously being primarily responsible for domestic work (Hochschild and Machung, 2012).

## Limitations and Future Directions

This review includes a selection of articles that are relevant to our specific hypothesis that exposure to or interaction with counterstereotypical role models reduce gender stereotyping and promote counterstereotypical aspirations and behavior. We conducted a thorough literature review, but not a systematic search due to counterstereotypical role models being variably defined in the literature. We selected literature that both confirmed and challenged our hypothesis, with the aim to produce a balanced narrative review. We encourage researchers to conduct a meta-analysis on the studies reviewed above to integrate role model effects more systematically. More research is also needed on whether exposure to counterstereotypical male role models influence boys’ and men’s gender stereotyping and career choices. Men are underrepresented in communal occupations and roles (Croft et al., 2015). However, very few field-based role model interventions have been implemented to promote communal behavior in boys and men. Whilst we assume that the same processes that underlie role model effects would apply for boys and girls, experimental research has produced inconsistent findings. Sometimes studies have found a role model effect for girls but not boys, and sometimes studies have found a role model effect for boys but not girls (Katz, 1986; Buren et al., 1993; Green et al., 2004; Pike and Jennings, 2005). Future research should investigate the reason for these mixed findings. On a final note, gender roles have changed over the last few decades. Thus, moving forward, more carefully designed research on the impact of counterstereotypical role models in early childhood and scientific evaluations of initiatives and interventions in adolescence are warranted in order to see whether previous findings replicate across time and contexts.

## Author Contributions

SM and MO conceived of the presented idea. MO reviewed the literature. SM supervised the findings of this work. Both authors discussed the results and contributed to the final manuscript.

## Conflict of Interest Statement

The authors declare that the research was conducted in the absence of any commercial or financial relationships that could be construed as a potential conflict of interest.
